# Reliability of technologies to measure the barbell velocity: Implications for monitoring resistance training

**DOI:** 10.1371/journal.pone.0232465

**Published:** 2020-06-10

**Authors:** Alejandro Martínez-Cava, Alejandro Hernández-Belmonte, Javier Courel-Ibáñez, Ricardo Morán-Navarro, Juan José González-Badillo, Jesús G. Pallarés

**Affiliations:** 1 Human Performance and Sports Science Laboratory, Faculty of Sport Sciences, University of Murcia, Murcia, Spain; 2 Faculty of Sport, Pablo de Olavide University, Seville, Spain; Universidade Federal de Mato Grosso do Sul, BRAZIL

## Abstract

This study investigated the inter- and intra-device agreement of four new devices marketed for barbell velocity measurement. Mean, mean propulsive and peak velocity outcomes were obtained for bench press and full squat exercises along the whole load-velocity spectrum (from light to heavy loads). Measurements were simultaneously registered by two linear velocity transducers T-Force, two linear position transducers Speed4Lifts, two smartphone video-based systems My Lift, and one 3D motion analysis system STT. Calculations included infraclass correlation coefficient (ICC), Bland-Altman Limits of Agreement (LoA), standard error of measurement (SEM), smallest detectable change (SDC) and maximum errors (MaxError). Results were reported in absolute (m/s) and relative terms (%1RM). Three velocity segments were differentiated according to the velocity-load relationships for each exercise: heavy (≥ 80% 1RM), medium (50% < 1RM < 80%) and light loads (≤ 50% 1RM). Criteria for acceptable reliability were ICC > 0.990 and SDC < 0.07 m/s (~5% 1RM). The T-Force device shown the best intra-device agreement (SDC = 0.01–0.02 m/s, LoA <0.01m/s, MaxError = 1.3–2.2%1RM). The Speed4Lifts and STT were found as highly reliable, especially against lifting velocities ≤1.0 m/s (Speed4Lifts, SDC = 0.01–0.05 m/s; STT, SDC = 0.02–0.04 m/s), whereas the My Lift app showed the worst results with errors well above the acceptable levels (SDC = 0.26–0.34 m/s, MaxError = 18.9–24.8%1RM). T-Force stands as the preferable option to assess barbell velocity and to identify technical errors of measurement for emerging monitoring technologies. Both the Speed4Lifts and STT are fine alternatives to T-Force for measuring velocity against high-medium loads (velocities ≤ 1.0 m/s), while the excessive errors of the newly updated My Lift app advise against the use of this tool for velocity-based resistance training.

## Introduction

The ability to develop force rapidly against a continuum of loads is a key factor in sport performance. To be able to objectively quantify and monitor the actual training load undertaken by athletes is a key issue in the design of effective, efficient and safer training programmes [1]. The use of the barbell movement velocity as the main variable, namely the velocity-based training (VBT), constitute a practical alternative to traditional percentage-based training using the one repetition maximum (1RM) to estimate relative loads [2–4]. VBT relies on technology to track the lifting velocity in real time and adjust the training load based on the resultant velocity data [2]. The VBT has important practical implications for the design and implementation of individual training plans. On one hand, coaches are provided with quantitative outcomes that can be used for multiple purposes, such as training autoregulation through the warm-up loads’ velocity assessment [2,4,5], determination of individualised load-velocity profiles [6] and the real-time neuromuscular fatigue monitoring [4,7]. On the other hand, practitioners receive instantaneous performance feedback about the actual velocity developed during each lift, which has been shown to produce greater adaptation and larger training effects [8]. Due to the number of advantages, the adoption of the VBT approach among professionals from different sport disciplines has been rising in recent years [9].

There is increasing evidences showing that VBT could be more effective than traditional training methods to decrease training stress and improve velocity-specific adaptations [10,11]. An optimal VBT prescription needs the use of reliable devices to accurately measure the barbell velocity for effectively managing the training load and maximize the adaptive responses. This requirement constitutes one of the main drawbacks of VBT since very small changes in velocity can represent decisive improvements or decrements in neuromuscular and functional performance [12–14]. As a consequence, there is an increasing number of available devices to measure the barbell velocity using a wide variety of technologies. This technological development has been accompanied by a parallel increase in studies attempting to examine the validity and reliability of emerging devices, including linear velocity transducers [15], linear position transducers [16,17] and optoelectronic systems [18]. While these technologies have been specifically designed to measure the barbell velocity, some authors have tested the validity of camera-based tools such as smartphones apps [19,20], inertial measurement units [21] or 3D motion analysis system (3DMA) [22] as alternatives.

Due to this increasing interest in testing the reliability of barbell velocity measurement devices, there is a need to clarify some methods commonly used that may limit the data interpretation and conclusions. Firstly, they wrongly used the Pearson’s correlation coefficient to determine the level of agreement between devices. Pearson’s correlation quantifies the relationship between scores, but does not provide any insight into systematic errors inherent in the measurement; thereby, an excellent correlation does not mean complete agreement between scores [23]. Secondly, most of the statements in favour to a given device reliability are based on Bland-Altman plots. Whereas the use of Bland-Altman analysis requires the interpretation of the magnitude of errors according to practical criteria and established acceptable levels of disagreement [24,25], only a few studies have based their findings on these criteria [26–28]. An interesting approach has been presented based on the changes in performance (% 1RM) produced by increments in the barbell velocity [26–28]. Previous studies describing the load-velocity relationship for different resistance training exercises performed in a Smith machine observed that changes between 0.05 to 0.10 m/s in bench press (BP) and full squat (SQ) would represent 5% 1RM improvement [2,12,14]. Based on these findings, to determine these gains in performance, one would require a device accurate enough to ensure that the changes are not produced by the error of the measurement but represent a real performance improvement (i.e., error < 0.05 or 0.10 m/s, at least). Hence, one could consider that a given device with errors above this limit would not be reliable enough for VBT purposes. However, only two studies have made recommendations on barbell velocity measurement devices based on practical criteria [26,28], which encourage further research in this direction.

Finally, while all the available devices are apparently reliable to measure velocity in heavy-load lifting (i.e., < 0.50 m/s), the VBT requires the identification of measurement errors across a spectrum of relative loads, including fast movements against moderate and light loads [21,26–28]. In particular, monitoring high velocities are important to assess changes in neuromuscular and functional performance due to the higher specificity in relationship with most sporting movements [29,30]. In this regard, there is no available data about the reliability of the Speed4Lifts, the STT 3DMA camera system and My Lift app during actions > 1.0 m/s for the BP and SQ exercises. Because some devices could present greater errors when monitoring lifts at higher velocities [27], it would be important to determine the magnitude of errors throughout the whole load-velocity spectrum, for instance, heavy (≥ 80% 1RM), medium (50% < 1RM < 80%) and light loads (≤ 50% 1RM). Furthermore, it would be of interest to determine if the errors would allow the detection of changes in performance according to practical acceptable criteria, such as the 5% 1RM approach [26–28].

Altogether, the aforementioned drawbacks make it difficult to extrapolate the results to the practice and may question the suitability of emerging technologies to provide objective and reliable measurements for VBT. In addition, it is essential to inform about the magnitude of errors from different velocity segments and across the entire loading spectrum (from heavy to light loads) to help strength and conditioning practitioners in establishing velocity thresholds according to the training plan and performance targets. Hence, the aim of this study was to provide insights about the best use of each device by conducting a comprehensive reliability and reproducibility analysis on four different technologies used in VBT to determine inherent technical errors (i.e., the agreement between two devices from the same model and brand) and compare their level of agreement/disagreement against a criterion device.

## Materials and methods

### Participants

Fifteen males volunteered to participate in this study (Mean ± SD: age 27.0 ± 3.8 years old, body mass 78.8 ± 7.6 kg, height 178.0 ± 6.3 cm). All participants were familiarised with the testing protocols and had previously participated in similar studies. No physical limitations or musculoskeletal injuries that could affect testing were reported. Participants signed a written informed consent form. The study was conducted according to the Code of Ethics of the World Medical Association (Declaration of Helsinki) and approved by the Bioethics Commission of the University of Murcia.

### Study design

Participants underwent two experimental sessions in random order, one for the BP and one for the SQ exercises, separated by 48 h of recovery. Participants completed a progressive loading test in a Smith machine. This test consisted in performing one repetition against eight increasing fixed loads ranging from 25 to 95 kg, with 10 kg increments and 5 min of recovery. Seven devices based on four different technologies were used to simultaneously measure the barbell velocity during the performance of each repetition. The magnitude of errors, levels of agreement and linear relationships between two devices from the same brand and model (intra-device agreement) as well as between any given device compared to a gold standard (inter-device agreement) were calculated in overall and for particular loading ranges uses in practical settings (≤ 50%, 50–80%, and ≥ 80% 1RM).

### Methodology

A description of the BP and SQ testing protocols has been previously detailed [12,14]. After a familiarization session. Participants performed one repetition against eight fixed loads (25, 35, 45, 55, 65, 75, 85 and 95 kg) at maximal intended velocity with 5 min of rest between, in two sessions separated by 48–72 h (one per exercise). To ensure that all participants were able to complete the entire protocol, they performed a 1RM test in both exercises two weeks before the experiment, achieving 99.9 ± 3.2 kg in BP and 100.6 ± 2.7 kg in SQ (1.28 ± 0.11, 1.29 ± 0.12 normalized per kg of body mass, respectively). The eccentric phase was performed at a controlled velocity (0.50–0.70 m/s) for standardization and security reasons. This protocol was implemented during the familiarization sessions with the aid of the real-time feedback provided by the T-Force System, so that the velocity could be adjusted to the required range during the eccentric phase for all participants during actual testing procedures. Feet and grip positions (shoulder width or slightly wider) were measured so that they could be reproduced on every lift.

### Measurement equipment and data acquisition

Seven single device units representatives of four different technologies (Table 1), were used to simultaneously measure or estimate the barbell velocity during the upward part of the lifts (i.e., concentric phase) for each repetition, as previously explained [26]. In summary: two linear velocity transducers, T-Force Dynamic Measurement System (Ergotech Consulting, Murcia, Spain), two linear position transducers, Speed4Lifts (v2.0, Speed4Lifts, Madrid, Spain), two smartphone video-based apps, My Lift (version 8.1 iOS), installed on two iPhone 5S units running iOS 12.2 (Apple Inc., California, USA) and a set of 3DMA with six cameras, STT (STT system, Basque Country, Spain) and specific software (v6.10, STT Systems, País Vasco, Spain). Three distinct velocity outcome measures were obtained from each device, when possible: mean velocity (MV, mean concentric velocity); mean propulsive velocity (MPV, mean velocity of the propulsive phase, defined as that portion of the concentric phase during which barbell acceleration is greater than acceleration due to gravity) and peak velocity (PV, maximum instantaneous velocity reached during the concentric phase).

**Table 1:** Technical characteristics of the devices under study.

Device	T-Force System	Speed4Lifts	STT	My Lift
Technology	Linear velocity transducer	Linear position transducer	3D Motion Analysis system	Smartphone app
Software version	3.60	1.41 (Android)	6.10	8.1 (iOS)
Direct outcome measures	Velocity; Time	Position; Time	Position; Time	Position; Time
Indirect outcome calculations	Distance; Acceleration; Force; Power	Distance; Velocity; Power	Distance; Velocity	Distance; Velocity
Sampling frequency	1000 Hz	100 Hz	100 Hz	60 Hz
Mechanic variables displayed by the software	Mean, peak and time to reach peak values for all direct and indirect outcomes, propulsive phase, estimated load (%1RM), 1RM prediction, number of repetitions, velocity loss (%), velocity alerts (visual and audio feedback)	Mean propulsive and peak velocity, mean power, range of motion, estimated load (%1RM), 1RM prediction, number of repetitions, velocity loss (%) inter and intra-set (visual and audio feedback)	Position-time curve in axis: x (lateral displacement), y (vertical displacement) and z (antero-posterior displacement)	Peak vertical and horizontal displacement, peak and mean vertical velocity, instantaneous velocity and time
External power supply required	No	No	Yes	No
Installation and calibration time before the first execution*	2.4 min	2.5 min	2.2 h	1.5 min
Time to obtain the measure after execution*#*	real time	real time	130 s	45 s
Number of lost repetitions per each 100 cases	0.8	0	1.7	0

*Estimation of mean installation and equipment calibration time spent for the performance of three consecutive repetitions.

#Mean time required to obtain the MV, MPV or PV outcome value from three repetitions performed against medium to high loads (> 50% 1RM).

**Fig 1:**
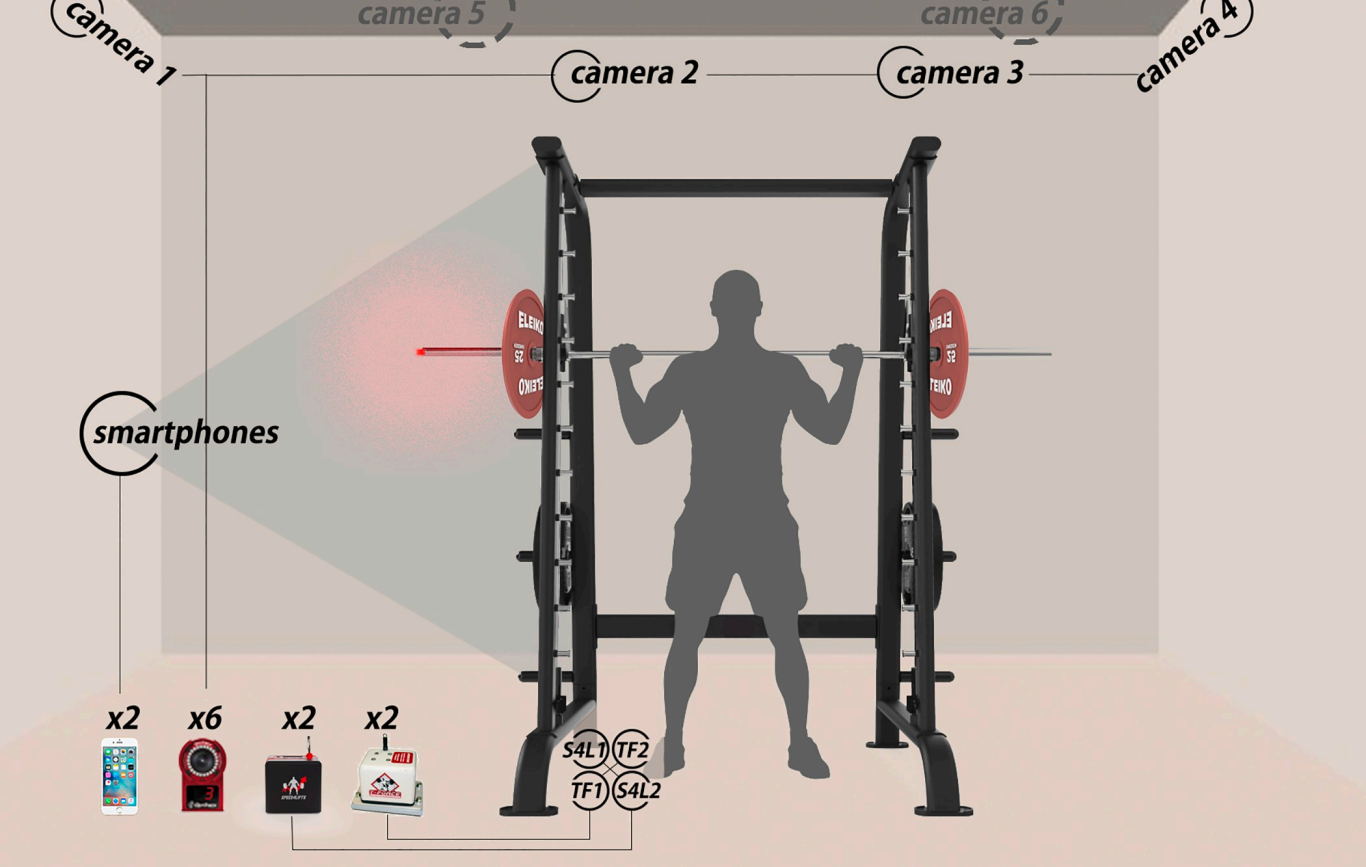
Experiment set up.

A visual representation of the experiment set up is shown in Fig 1. To avoid the appearance of errors due to the location of the devices [31], the retractable cables of all T-Force and Speed4Lifts units were attached to the same right side of the Smith Machine, all of them placed very close to the vertical displacement axis (3 cm to the right and left side of the axis), using a purpose-built support (Fig 1). None of the participants felt difficult or uncomfortable when lifting with the four retractable cables attached on one side of the barbell. The smartphones running the My Lift app were placed on tripods, at a horizontal distance of 2.4 m, just in the same lateral side where the other devices were located. The height of tripods was adapted in each exercise (BP: 1.0 m and SQ: 1.35 m) to track the whole movement. An experienced examiner used the automatic tracking mode available in the My Lift app following the developer´s instructions. Intra-examiner reliability was conducted to ensure the consistency of the outcomes. The STT camera-based system was synchronized to follow a retro-reflective marker (14 mm; B&L Engineering, Santa Ana, CA) placed on the centre of the end-cap, at the end of the barbell sleeves, in the same side of the barbell where the other devices were installed. Position and time raw data were collected to obtain the outcome variables. The start (y_1_) and end (y_2_) positions of the concentric phase were located and displacement of this phase was calculated as y_2_ –y_1_. Then, MV was obtained dividing the displacement of the concentric phase by the time required to complete it (data time each 0.01 second; i.e. 100 Hz sampling). Instantaneous velocity was calculated by the differentiation of the displacement data with respect to time. Finally, PV was defined as the highest value of instantaneous velocity during the concentric phase. Each device was assembled and calibrated according to the manufacturer's specifications before each session.

Intra-device reproducibility was assessed by comparing the velocity outcomes for the same trial simultaneously obtained by each pair of the same brand and model devices. Since only one set of STT was used, the intra-device analysis of this technology could not be examined. The technology with the best intra-device agreement was taken as the reference to assess the inter-device agreement of one representative unit of each technology.

### Statistical analysis

Reliability analyses included the calculation of a set of statistics aimed at providing information about the level of agreement and the magnitude of errors (both in absolute and relative values) incurred when using the different technologies under study. To determine the magnitude of errors at particular velocity ranges [27], data were then classified on three velocity segments according to the velocity-load relationships for each exercise [12,13]: heavy loads (≥ 80% 1RM, MPV ≤ 0.50 m/s in BP and 0.70 m/s in SQ), medium loads (50% < 1RM < 80%, MPV between 0.50 and 1.00 m/s in BP and between 0.70 and 1.15 m/s in SQ) and light loads (≤ 50% 1RM, MPV ≥ 1.00 m/s in BP and ≥ 1.15 m/s in SQ). A detailed explanation of the statistical analyses conducted has been described elsewhere [26,27]. Correlation analyses included the Pearson’s correlation coefficient (*r*), the intraclass correlation coefficient (ICC, one way-random, absolute agreement) and the Lin’s concordance correlation coefficient (CCC), considering values over 0.99 as an almost perfect concordance, over 0.95 as moderate concordance and below 0.90 as a poor concordance [32]. Linear regression analyses were used to provide predictive equations for each device and calculate the standard error of the estimate (SEE). The standard error of measurement (SEM) was calculated from the square root of the mean square error term in a repeated-measures ANOVA to determine the variability caused by measurement error [33]. Data were presented in absolute (m/s) and relative terms as a coefficient of variation (CV = 100 SEM/mean). The smallest detectable change (SDC) was calculated from the SEM (SCD = √2×SEM×1.96) and considered as the change in the instrument score beyond measurement error [34]. Bland-Altman plots were used to assess and display the agreement along the entire spectrum of loads and at each velocity segment. Systematic difference (bias) and its 95% limits of agreement (LoA = bias ± 1.96 SD) were calculated. Maximum errors (MaxError) were calculated from the SEE (Max Error_SEE_) and the bias (MaxError_bias_) as the double of the upper bound of a 95% CI to represent the largest error expected from a given measurement [26] and were expressed in absolute values (m/s) and as the corresponding relative load (% 1RM) for each velocity and exercise based on previous studies [2,12,14]. Criteria for acceptable reliability were ICC > 0.990 and SDC < 0.07 m/s (~5% 1RM) according to previous recommendations [26,28,32] and based on the differences identified in MPV after short-term resistance training interventions [3,35,36].

## Results

**Fig 2:**
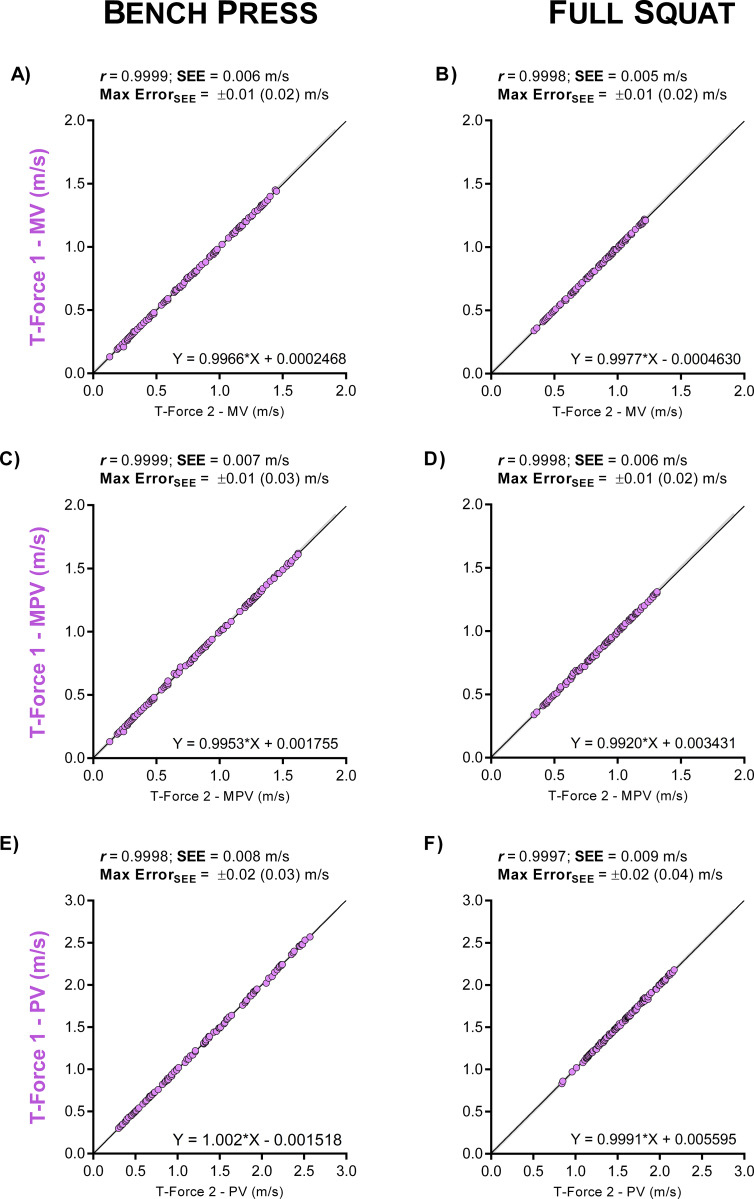
Intra-device agreement between two T-Force devices. Linear regressions for the velocity readings in bench press (A, C and E panels) and full squat (B, D and F panels) exercises. Panels are ordered by velocity outcomes: mean velocity (MV), mean propulsive velocity (MPV) and peak velocity (PV).

**Fig 3:**
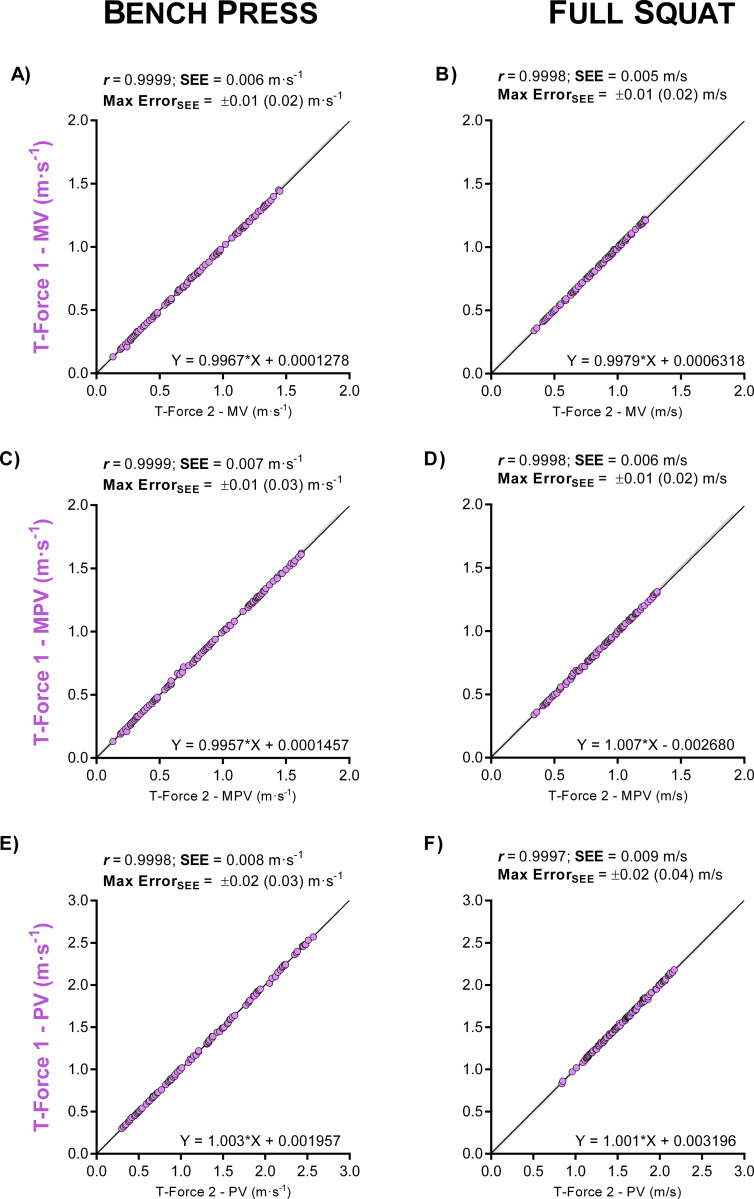
Intra-device agreement between two T-Force devices. Bland–Altman plots for the velocity readings in bench press (A, C and E panels) and full squat (B, D and F) exercises. Panels are ordered by velocity outcomes: mean velocity (MV), mean propulsive velocity (MPV) and peak velocity (PV). The grey shaded area indicates an acceptable level of agreement between devices, which results in differences in terms of load ≤ 5% 1RM [26,27].

Results from intra- and inter-device agreements for BP and SQ exercises are shown in Table 2. Intra-device comparisons showed the T-Force (Figs 2 and 3) as the most reproducible device (ICC and CCC = 1.000, CV ≤ 0.62%, SEM ≤ 0.01 m/s, SDC ≤ 0.02 m/s). The second-best intra-device results were obtained with the Speed4Lifts (ICC ≥ 0.999, CCC ≥ 0.999, CV ≤ 1.80%, SEM ≤ 0.02 m/s, SDC ≤ 0.05 m/s). My Lift showed the greatest errors and the worst reproducibility (ICC ≥ 0.972, CCC ≥ 0.945, CV ≥ 5.79%, SEM ≥ 0.08 m/s, SDC ≥ 0.24 m/s), despite the high intra-examiner agreement observed in the BP (ICC ≥ 0.998, CCC ≥ 0.997, SEM ≤ 0.04, CV ≤ 2.9%) and SQ (ICC ≥ 0.981, CCC ≥ 0.959, SEM ≤ 0.06 CV ≤ 3.9%).

**Table 2:** Intra- and inter-device agreement obtained for the velocity outcomes in the bench press and full squat exercise.

	Bench press (BP)						Full Squat (SQ)					
	Intra-device agreement			Inter-device agreement*			Intra-device agreement			Inter-device agreement*		
	*T-Force*	*Speed4Lifts*	*My Lift*	*Speed4Lifts*	*My Lift*	*STT*	*T-Force*	*Speed4Lifts*	*My Lift*	*Speed4Lifts*	*My Lift*	*STT*
*Peak velocity (PV)*												
SEM *m/s*	0.01	0.02	0.08	0.06	0.10	0.08	0.01	0.01	0.08	0.02	0.12	0.07
SDC *m/s*	0.02	0.05	0.23	0.18	0.26	0.21	0.02	0.04	0.24	0.07	0.34	0.19
CV *%*	0.45	1.54	5.79	4.94	7.04	5.57	0.46	0.86	5.02	1.60	7.59	4.22
Max Error *% 1RM*	1.8	4.4	25.0	15.7	19.4	10.4	2.2	4.3	28.1	7.0	24.3	9.7
ICC	1.000	1.000	0.993	0.995	0.991	0.994	1.000	0.999	0.972	0.997	0.937	0.981
CI-95% lower	1.000	0.999	0.990	0.993	0.987	0.991	1.000	0.999	0.959	0.996	0.910	0.973
CI-95% upper	1.000	1.000	0.995	0.997	0.993	0.996	1.000	0.999	0.980	0.998	0.956	0.987
CCC	1.000	0.999	0.986	0.991	0.981	0.988	1.000	0.998	0.945	0.994	0.890	0.963
*Mean propulsive velocity (MPV)*												
SEM *m/s*	0.01	0.02	-	0.02	-	-	0.01	0.01	-	0.03	-	-
SDC *m/s*	0.01	0.04	-	0.06	-	-	0.01	0.03	-	0.08	-	-
CV *%*	0.62	1.80	-	2.72	-	-	0.58	1.24	-	3.09	-	-
Max Error *% 1RM*	1.8	4.9	-	7.8	-	-	1.8	4.3	-	9.5	-	-
ICC	1.000	0.999	-	0.999	-	-	1.000	0.999	-	0.995	-	-
CI-95% lower	1.000	0.999	-	0.998	-	-	1.000	0.999	-	0.994	-	-
CI-95% upper	1.000	1.000	-	0.999	-	-	1.000	0.999	-	0.997	-	-
CCC	1.000	0.999	-	0.997	-	-	1.000	0.999	-	0.991	-	-
*Mean velocity (MV)*												
SEM *m/s*	< 0.01	-	-	-	-	0.03	< 0.01	-	-	-	-	0.01
SDC *m/s*	0.01	-	-	-	-	0.08	0.01	-	-	-	-	0.04
CV *%*	0.55	-	-	-	-	3.34	0.44	-	-	-	-	1.61
Max Error *% 1RM*	1.4	-	-	-	-	4.1	1.0	-	-	-	-	4.5
ICC	1.000	-	-	-	-	0.998	1.000	-	-	-	-	0.999
CI-95% lower	1.000	-	-	-	-	0.997	1.000	-	-	-	-	0.998
CI-95% upper	1.000	-	-	-	-	0.998	1.000	-	-	-	-	0.999
CCC	1.000	-	-	-	-	0.995	1.000	-	-	-	-	0.995

*The reference for assessing inter-device agreement was considered to be the device with the best intra-device agreement: T-Force (Figs 1 and 2). SEM: standard error of measurement; SDC: smallest detectable change; CV: SEM expressed as a coefficient of variation; Max Error: maximum error in %1RM calculated from the Bland-Altman bias; ICC: intraclass correlation coefficient; CI: confidence interval; CCC: Lin’s concordance correlation coefficient.

**Fig 4:**
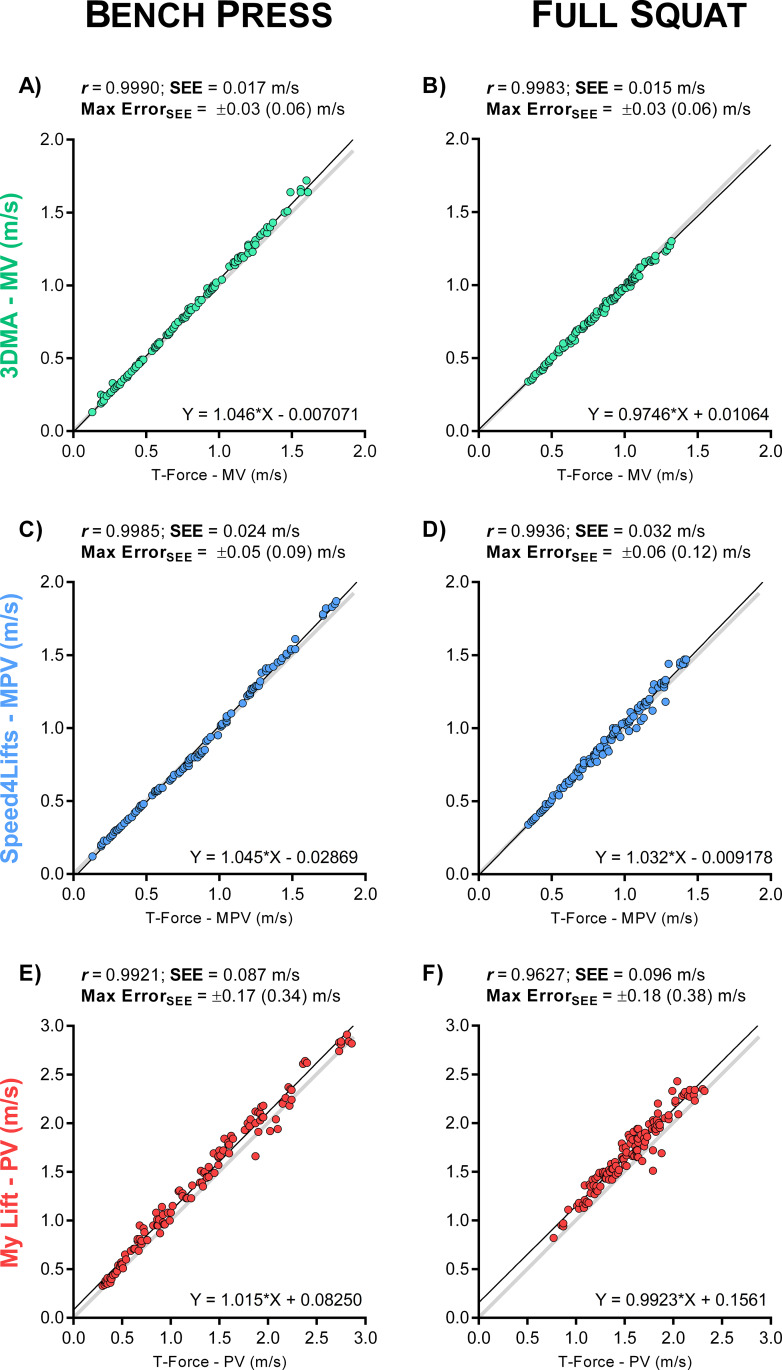
Linear regression analyses for the inter-device agreement in bench press (BP) and full squat (SQ) exercises. Each technology is presented in a different colour and compared against the reference.

**Fig 5:**
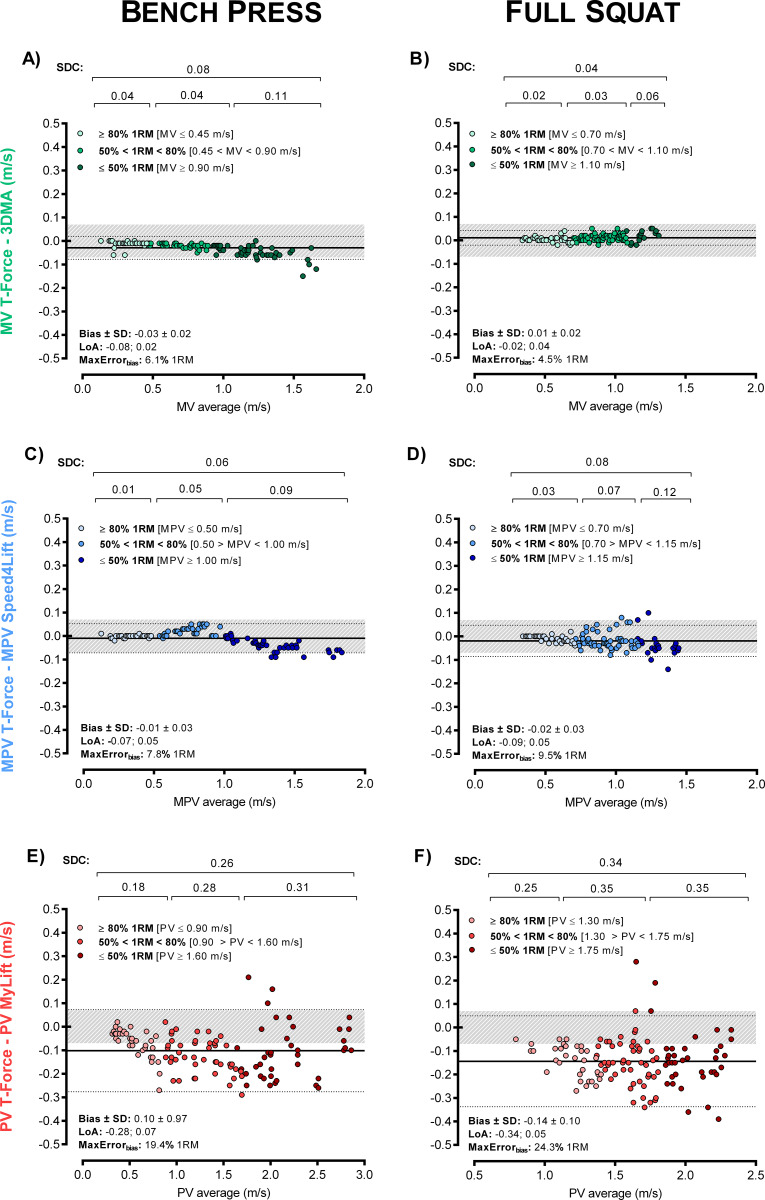
Bland-Altman plots for the inter-device agreement in bench press (BP) and full squat (SQ). Each technology is presented in a different colour and compared against the reference. The grey shaded area indicates an acceptable level of agreement between devices, which results in differences in terms of load ≤ 5% 1RM [26,27].

Inter-device linear regression analyses (Fig 4) and Bland-Altman plots (Fig 5) for the three different velocity segments identified the readings from STT and the Speed4Lifts as the most similar to the criterion device (T-Force), especially against medium to heavy loads (i.e., mean lifting velocities < 1.0 m/s). However, both the STT and the Speed4Lifts showed greater errors as the velocity increased. For instance, the SDC for Speed4Lifts at MPV ≤ 0.5 m/s were 0.01 and 0.02 m/s for BP and SQ respectively (Fig 4C and 4D) but increased up to 0.09 and 0.10 m/s for MPV > 1.0 m/s. The fact that a given device may produce greater errors when monitoring higher velocities, even if it shows reliable measures at low velocities (e.g., 1RM), has been previously suggested [27]. Moreover, the Speed4Lifts showed a changing trend, going from a slight underestimation for slow velocities to an over-estimation of the MPV for high velocities in the BP exercise compared to the T-Force (Fig 4C and 4D). My Lift showed the worst reproducibility and the highest errors in both BP and SQ exercises, regardless of velocity (Fig 4E and 4F). All the devices showed the smallest errors at slow velocities (< 0.50 m/s).

## Discussion

Based on the results of the present study, the linear velocity transducer T-Force stands as the most reliable technology for VBT purposes, showing the finest readings among the tested devices. The linear position transducer Speed4Lifts and the STT camera-based system were found as suitable alternatives to the T-Force, especially when monitoring movements against medium to heavy loads (Table 2). Nonetheless, our results suggest considering specific margins of errors for each exercise (BP or SQ), velocity parameter (MV, MPV and PV) and load spectrum (from heavy loads at slow velocities to light loads at high velocities) according to the SEM and SDC values obtained. Assuming the SDC as a change beyond measurement error [34], coaches and practitioners using a particular device should take these values as a confidence interval to make load adjustments, determine the number of repetitions and identify training adaptations. Otherwise, it might be possible that the increments in the velocity come from a measurement error and therefore the load adjustments could produce adverse effects and increase the risk of injury, illness or overtraining [1]. Whereas this is the first time testing the intra-device agreement of the Speed4Lifts, our findings support the only previous study examining the reliability of this tool [17]. These authors observed a high agreement between the Speed4Lifts readings and the Trio-OptiTrack 3DMA system when measuring BP and SQ lifts from 0.38 to 0.88 m/s. Our study adds to this previous research by noting that Speed4Lifts reliability decreases as the velocity movement increases. In particular, we identify increments in MPV errors up to 0.05/0.07 m/s for BP/SQ at 50–80% 1RM (Fig 5C). This is an important limitation for the Speed4Lifts to monitor resistance training against light-loads (high-velocities) and explosive movements. All in all, the Speed4Lifts is one of the most affordable devices on the market and it may be considered as an adequate and practical tool for VBT, notwithstanding the aforementioned observations.

The STT showed excellent results in the MV variable against medium to heavy loads, but greater errors for the fastest movements (Fig 5A and 5B). One recent study has tested the reliability of a similar 3DMA system (Qualisys Track Manager) to assess the barbell velocity with similar findings [22]. The worse performance of the 3DMA system to monitor high-velocity lifts could be attributable to the limited sampling rate of the cameras (i.e., the faster the movement, the shorter the time and the lower the number of data points, resulting in greater errors). Likewise, it was not possible to accurately estimate the MPV since the end of the propulsive phase during resistance training exercises lasts less than 0.01 seconds [15]. Nonetheless, assuming that technological advancements may solve this limitation in the future by developing faster cameras, several important disadvantages of 3DMA systems, such as expensiveness of the equipment and time-consuming setup and data processing, makes it unpractical in real-world settings.

The My Lift smartphone app (formerly PowerLift) has attracted much attention due to its low cost, versatility and ease of implementation [17,19,20]. The new update of the app includes an automatic tracking mode that estimates velocity from a side-view video by manually setting the diameter of the weight discs as a reference. Despite the increasing popularity of smartphone apps for VBT, our results showed that the My Lift was the least reliable tool compared to the other available devices (Fig 5E and 5F). These results are consistent with previous research [19,27,37] suggesting that My Lift might be only reliable to track slow lifts with heavy loads. Although this might be useful to conduct routinely submaximal loading tests at > 80% 1RM and to avoid reaching the 100%, the use of the My Lift app for VBT is ill-advisable due to its large measurement errors (SEM > 0.10 m/s, SDC > 0.23 m/s) when tracking lifts > 0.30 m/s, making it difficult to determine load adjustments or performance fluctuations with sufficient accuracy. It must be noted that we found a very high and consistent intra-examiner reliability, meaning that the app is user-friendly and confirming that the results were not affected by the examiner’s handling but the device itself. At the time of this study, no previous research has examined the reliability of this new update of the My Lift app, which encourages further investigations.

The list of statistical calculations provided herein represents an added value compared to the majority of studies testing the validity and reliability of velocity measurement devices for resistance training. Practitioners should consider at least the SEM as the limit below which a given device should be used, although the SDC would be advisable to identify meaningful changes in performance and determine the real effort being incurred during training. In this paper, we provide the SDC values for different segments (slow, medium and high velocities) along a broad range of velocities (MV > 0.2 to < 2.8 m/s). Furthermore, it has been demonstrated that traditional interpretation of correlations and linear relationship coefficients (i.e., values >0.90 as very high) failed to determine the reliability of a device [24–26]. As could be expected, all the devices in the present study showed high r and ICC values ~0.99, with 0.937 in the worst case. This just means that the faster the lift, the higher the measure of the device but gives no information about the magnitude of errors in absolute values. Given that practitioners make load adjustments in absolute values (i.e., m/s), the CCC appears to be a more appropriate coefficient than the ICC and *r* to determine the reliability of VBT devices. Additionally, the use of Bland-Altman bias requires the interpretation of the results using acceptable limits of agreement based on practical criteria. The recommendations provided in our study are tailored to VBT practitioners and based on specific margins of errors previously defined [26]. We encourage future researchers to follow this approach in order to assist coaches and practitioners in the use of velocity measurements to decide which device to choose and thus provide better training prescription.

Strength and conditioning practitioners, and particularly those using VBT, should consider the magnitude of errors for their preferable device as a confidence interval to make load adjustments, determine the number of repetitions and identify training adaptations with sufficient accuracy. Otherwise, it might be possible that the increments in the velocity came from a measurement error and therefore the adjustments could produce adverse training adaptations and increase the risk of injury. Our findings suggest coaches and researchers to use the T-Force device as preferable option for monitoring barbell velocity and identifying technical errors of measurement for emerging devices. If T-force device is unavailable, both the Speed4Lifts and the STT system can be used as a highly reliable option, especially against velocities ≤1.0 m/s. Finally, practitioners are discouraged to use the automatic tracking mode of My Lift app for assessing barbell velocity, since its high errors are well above the acceptable levels.

This work has some important strengths, such as the interpretation of the magnitudes of errors according to practical criteria (i.e., 5% 1RM) and the identification of particular measurement errors for light, medium or heavy loads. The current recommendations by segments may serve as a practical guide to assist coaches and practitioners in the election of one device or another depending on their practical interests. It also seems to be the first study examining the reliability of the Speed4Lift, the My Lift app and the STT 3DMA system during the BP and SQ at lifts > 1.0 m/s. Although the present paper has not tested the biological variability purposely but determine the technological error, there are available studies examining the changes in the measures during repeated trials in common resistance training exercises [6,26,38]. The choice of one technology over another should be taken according to the particular context and depending on the accuracy required to identity true changes in performance.

## Conclusions

Taken together, our findings suggest that the linear velocity transducer is an extremely reliable technology for VBT purposes with the T-Force showing the finest readings among the tested devices along the entire spectrum of velocities (MV > 0.2 to < 2.8 m/s). The linear position transducer Speed4Lifts and the STT camera-based system are suitable alternatives to the T-Force, especially to monitor barbell movements against medium to heavy loads (< 1.0 m/s). On the other hand, the My Lift smartphone app (formerly PowerLift) is ill-advisable for VBT due to its large measurement errors when tracking lifts > 0.30 m/s.
