# Correction: Reliability of technologies to measure the barbell velocity: Implications for monitoring resistance training

**DOI:** 10.1371/journal.pone.0236073

**Published:** 2020-07-09

**Authors:** Alejandro Martínez-Cava, Alejandro Hernández-Belmonte, Javier Courel-Ibáñez, Ricardo Morán-Navarro, Juan José González-Badillo, Jesús G. Pallarés

Fig 3 is incorrect. The authors have provided a corrected version here.

**Fig 3:**
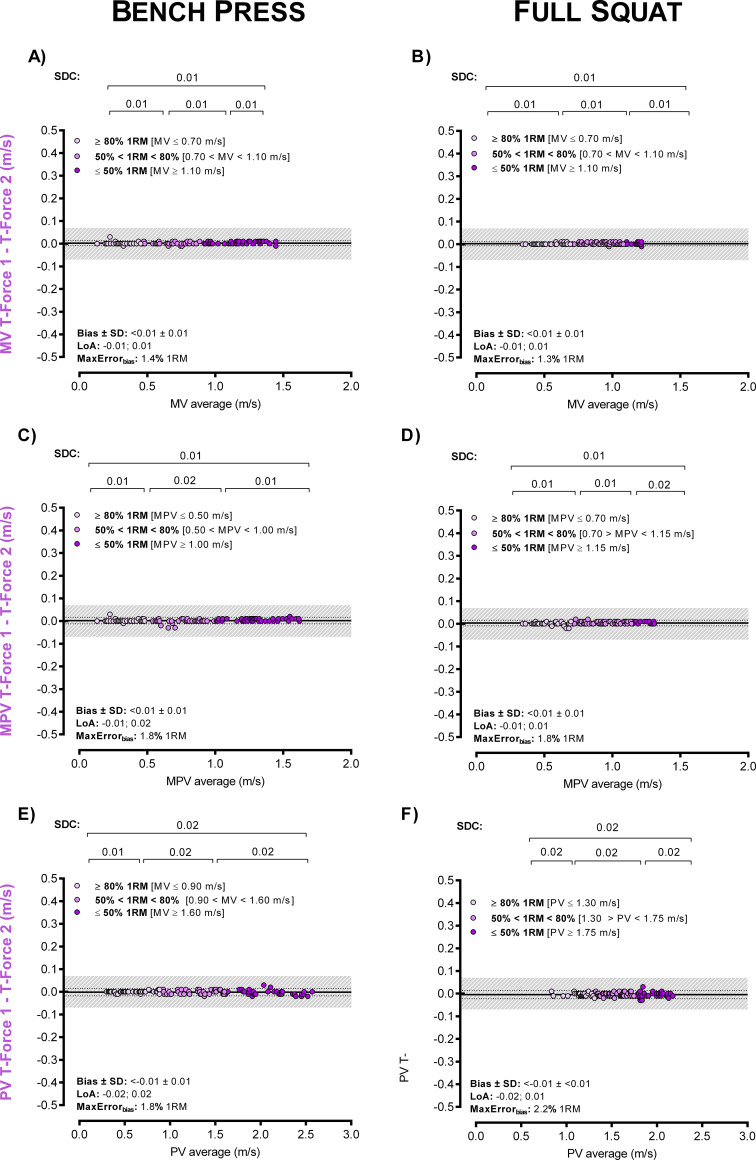
Intra-device agreement between two T-Force devices. Bland–Altman plots for the velocity readings in bench press (A, C and E panels) and full squat (B, D and F) exercises. Panels are ordered by velocity outcomes: mean velocity (MV), mean propulsive velocity (MPV) and peak velocity (PV). The grey shaded area indicates an acceptable level of agreement between devices, which results in differences in terms of load ≤ 5% 1RM [26,27].
